# The Uncoordinated-5 Homolog B (UNC5B) Receptor Increases Myocardial Ischemia-Reperfusion Injury

**DOI:** 10.1371/journal.pone.0069477

**Published:** 2013-07-23

**Authors:** David Köhler, Ariane Streißenberger, Klemens König, Tiago Granja, Judith M. Roth, Rainer Lehmann, Claudia Bernardo de Oliveira Franz, Peter Rosenberger

**Affiliations:** 1 Department of Anesthesiology and Intensive Care Medicine, University Hospital, Tübingen, Germany; 2 Department of Internal Medicine, Division of Clinical Chemistry, University Hospital, Tübingen, Germany; University of Colorado Denver, United States of America

## Abstract

The UNC5 receptor family are chemorepulsive neuronal guidance receptors with additional functions outside the central nervous system. Previous studies have implicated that the UNC5B receptor influences the migration of leukocytes into sites of tissue inflammation. Given that this process is a critical step during the pathophysiology of myocardial ischemia followed by reperfusion (IR) we investigated the role of UNC5B during myocardial IR. In initial in-vitro experiments, the functional inhibition of UNC5B resulted in a significant reduction of chemotactic migration of neutrophils. In-vivo, using a model of acute myocardial ischemia in *UNC5B^+/−^* and wild type (WT) animals, we found a significant reduction of infarct sizes in *UNC5B^+/−^* animals. This was associated with significantly reduced levels of troponin-I and IL-6 in *UNC5B^+/−^* mice. The repression of UNC5B using siRNA and the functional inhibition of UNC5B significantly dampened the extent of myocardial IR injury. Following depletion of neutrophils, we were not able to observe any further reduction in infarct size through functional inhibition of UNC5B in WT and *UNC5B^+/−^* mice. In summary our studies demonstrate an important role for UNC5B during myocardial IR injury, and that UNC5B might be a potential therapeutic target to control reperfusion injury in the future.

## Introduction

Ischemic heart disease is amongst the leading causes for morbidity and mortality worldwide, and early reperfusion of the infarcted area is to date the treatment of choice [1]. This reperfusion reduces the size of damaged myocardial tissue and improves clinical outcome of affected individuals. Nevertheless, reperfusion of the ischemic myocardium can also induce injury of the affected tissue. This phenomenon, termed myocardial ischemia-reperfusion (IR) injury, paradoxically reduces the beneficial effects of reperfusion. Hallmarks of the reperfusion phase are cellular swelling, contracture of myofibrils, disruption of the sarcolemma and the infiltration of leukocytes into the ischemic tissue. This structural derangement is at least in part caused by neutrophils, which are attracted to the ischemic tissue [2], [3].

It is well established that activation and migration of leukocytes is controlled through the chemokine system. However recent studies provide evidence that neuronal guidance proteins (NGP) and their receptors display an alternative class of guidance cues in the immune system that steer immune responses particularly with regard to activation and the migration of leukocytes. NGP were first identified in the developing central nervous system (CNS), where neurons and axons are precisely guided to their final location by a balance of chemoattractive and chemorepulsive signals to establish the elaborate neuronal circuitry. Several families of such conserved neuronal guidance cues influencing axonal migration were identified to date. Recent data provide evidence that the NGP receptor Uncoordinated-5 homolog B (UNC5B) also holds additional function outside the nervous system especially in the control of the immune system [4]–[7]. The endogenous ligand of UNC5B, the NGP netrin-1 has shown potent anti-inflammatory properties in animal models of hypoxia, ventilator associated lung injury, peritonitis and renal ischemia-reperfusion injury. Ly et al. have provided evidence that UNC5B itself is a crucial receptor involved into the chemotactic transmigration of immune competent cells, inhibiting further inflammation of affected tissues [8]. Furthermore, the importance of UNC5B during IR injury has been evaluated in previous work of our group during hepatic IR injury [9].

Given these previously known properties of UNC5B we suggested that UNC5B might hold significant impact on the pathophysiology of myocardial IR injury. We report here that UNC5B is expressed in murine organs abundantly outside the CNS. The inhibition of UNC5B receptor substantially affects the migration of neutrophil granulocytes (PMNs) in vitro. These results translated into significantly reduced myocardial injury and significantly reduced PMN infiltration in *UNC5B^+/−^* mice compared to WT controls. Furthermore, the repression of UNC5B through siRNA, the inhibition through anti-UNC5B antibody and the depletion of neutrophils demonstrated a significant importance of UNC5B for the extent of myocardial ischemia reperfusion injury.

## Materials and Methods

### Ethic Statement

All animal protocols were in accordance with the German guidelines for use of living animals and were approved by the Institutional Animal Care and Use Committee of the Tübingen University Hospital and the Regierungspräsidium Tübingen. UNC5B+/− mice were generated, validated and characterized as described previously (4). WT controls (C57BL/6J mice) were bred as littermates of the UNC5B+/− mice. After approval by the Institutional Review Board of Tübingen University Hospital and after written informed consent was obtained from each person studied blood was withdrawn and PMN isolated from whole blood obtained. PMNs isolated from human blood was used in transendothelial migration and paracellular flux assays. The withdrawal was approved by the Institutional Review Board of Tübingen University Hospital (ethics committee). The blood donor participants provided their written consent and were registered accordingly.

### Murine Model of Myocardial Ischemia

*UNC5B^+/−^* mice and controls were selected to be similar in age-, gender- and weight. After anesthesia was induced animals were placed on a temperature-controlled heated table to maintain body temperature at 37°C. Animals were orally intubated and ventilated (Servo 900C, Siemens, Germany). Following left parasternal thoracotomy, the left coronary artery (LCA) was visually identified, an 8.0 nylon suture (Prolene, Ethicon) was placed around the vessel and ischemia induced using a model described previously [10]. Infarct sizes were determined by calculating the percentage of myocardial infarction compared to the area at risk (AAR) using double staining technique with Evan’s blue and triphenyltetrazolium chloride (TTC) [11]. AAR and the infarct size were determined via planimetry using NIH software ImageJ 1.44p and the degree of myocardial damage was calculated as percentage of infarcted myocardium from the AAR.

### In vivo Small Interfering (si) RNA Repression

To achieve in vivo repression of UNC5B we used 2.5 µg/g body weight UNC5B ON-TARGETplus SMARTpool mouse siRNA dissolved in 5% Glucose-solution. siRNA was given intravenously 24 hours before ischemia and control non-targeting siRNA (Thermo Scientific) with at least four mismatches to any human, murine or rat gene was used. The siRNA target sequences of the UNC5B pool were, J-050737-09 with target sequence CCU ACG UAU UCA UGG GCG A, J-050737-10 with target sequence CCG UGG GAG UGA UCG UAU A, J-050737-11 with target sequence CGG CCA CAG UCA UCG UCU A, J-050737-12 with target sequence CGG AGA AAC UGC CGG GAC U. As transfection reagent in vivo-jetPEI (Polyplus transfection) was used.

### In vivo UNC5B Functional Blockade with Anti-UNC5B Antibody

To achieve functional inhibition of UNC5B we administered 2.5 µg anti-UNC5B antibody per mouse (R&D Systems) antibody dissolved in 100 µl PBS via tail vein injection, 30 min before surgery intervention. As control an IgG antibody (R&D Systems) dissolved in 100 µl PBS was used.

### In vivo Depletion of Murine Neutrophils

In a subset of myocardial ischemia experiments PMN depletion was achieved by neutrophil specific antibody treatment (RB6-8C5, Ly-6G 150 µg per mouse; BD Bioscience) dissolved in 100 µl saline via tail vein injection, 20 hours before surgery. 30 min before surgical intervention we additionally administered anti-UNC5B antibody for functional blocking, like described above.

### Western Blots for UNC5B

WT and *UNC5B^+/−^* mice were sacrificed and relevant organs harvested. After homogenization tissues were centrifuged and the resulting pellet was resuspended in RIPA buffer. Polyclonal rabbit anti-UNC5B antibody was used (sc-98865; Santa Cruz Biotechnology). Loading conditions were controlled by a rabbit polyclonal GAPDH antibody (Santa Cruz Biotechnology). Detection was achieved by application of alkaline phosphatase (Santa Cruz Biotechnology).

### Isolation of Human PMNs, Transendothelial Migration and Paracellular Flux Assay

PMNs were isolated from human peripheral whole blood. For transendothelial migration studies, HMEC cells were grown on a permeable membrane of transwell inserts (3 µm pore size) until confluent. In subsets of experiments PMNs or endothelium were pre-incubated with UNC5B antibody (R&D Systems) or IgG control. As chemoattractant 10 ng/ml fMLP (N-formyl-methionine-leucine-phenylalanine) in HBSS^+^ was added to the lower compartment of the transwells. Transmigration of PMNs was quantified as described previously [12].

For measuring paracellular flux FITC-labeled dextran (70 kDa) was added into the apical compartment and flux determined as described previously [13].

### Flowcytometry Analysis of UNC5B Expression

Human whole blood from healthy volunteers was collected and incubated with following antibodies (a) PE labeled: mouse IgG1, IgG2a, IgM (b) PerCP labeled: mouse IgG1 and (c) FITC labeled: mouse IgG2b (all BD Bioscience). As detection antibodies we used: anti-UNC5B (Abcam) FITC labeled, PE-mouse anti-human for CD3+ CD19; CD14 and CD15 detection and PerCP-mouse anti-human for CD45 detection (all BD Bioscience). The samples were evaluated with a BD FACS Calibur B0520 unit using BD CellQuestPro Software.

### Immunofluorescence Staining of Different Leukocytes

Blood was collected and after preparation following antibodies were used for labelling: anti-UNC5B (SC-98865); anti-CD45 (SC-25590); anti-CD15 (SC-19648); anti-CD14 (SC-73795) (all from Santa Cruz Biotechnology); anti-CD3 (MCA 463GT), and anti-CD19 (MCA 194OT) both from AbD Serotec Germany. As second antibody we used an Alexa 594-conjugated goat anti-mouse IgG (Invitrogen) for blood cell type identification. A goat anti-rabbit secondary antibody Alexa 488 conjugated (Invitrogen) was used to visualize UNC5B. Following the staining protocol cells were mounted and Axiophor Zeiss microscope (Zeiss, Jena) was used for imaging.

### Immunofluorescence Staining of Neutrophils in Tissue Sections

Untreated and ischemic hearts (60 min) of WT and *UNC5B^+/−^* mice were excised and immediately frozen. Cryostat sections of the hearts were stained with a rat anti mouse Ly-6B.2 Alloantigen (Monoclonal antibody; clone 7/4; isotype IgG2a, an anti-neutrophil antibody (AbD Serotec). As second antibody we used an Alexa 594-conjugated chicken anti-rat IgG (Invitrogen) and an anti-troponin antibody (sc-15368, Santa Cruz Biotechnology). Goat anti-rabbit secondary antibody Alexa 488 conjugated (Invitrogen) was used to visualize troponin. Fluorescence imaging was performed using Axiophot Zeiss microscope (Zeiss; Jena) with an AxioVision 4.8 software.

### Data Analysis

We performed statistical analysis using one-way analysis of variance (ANOVA) to determine group difference using post-hoc analysis followed by unpaired Student *t* test. A value of P<0.05 was considered to be statistically significant.

## Results

### UNC5B Expression Outside the Nervous System

**Figure 1:**
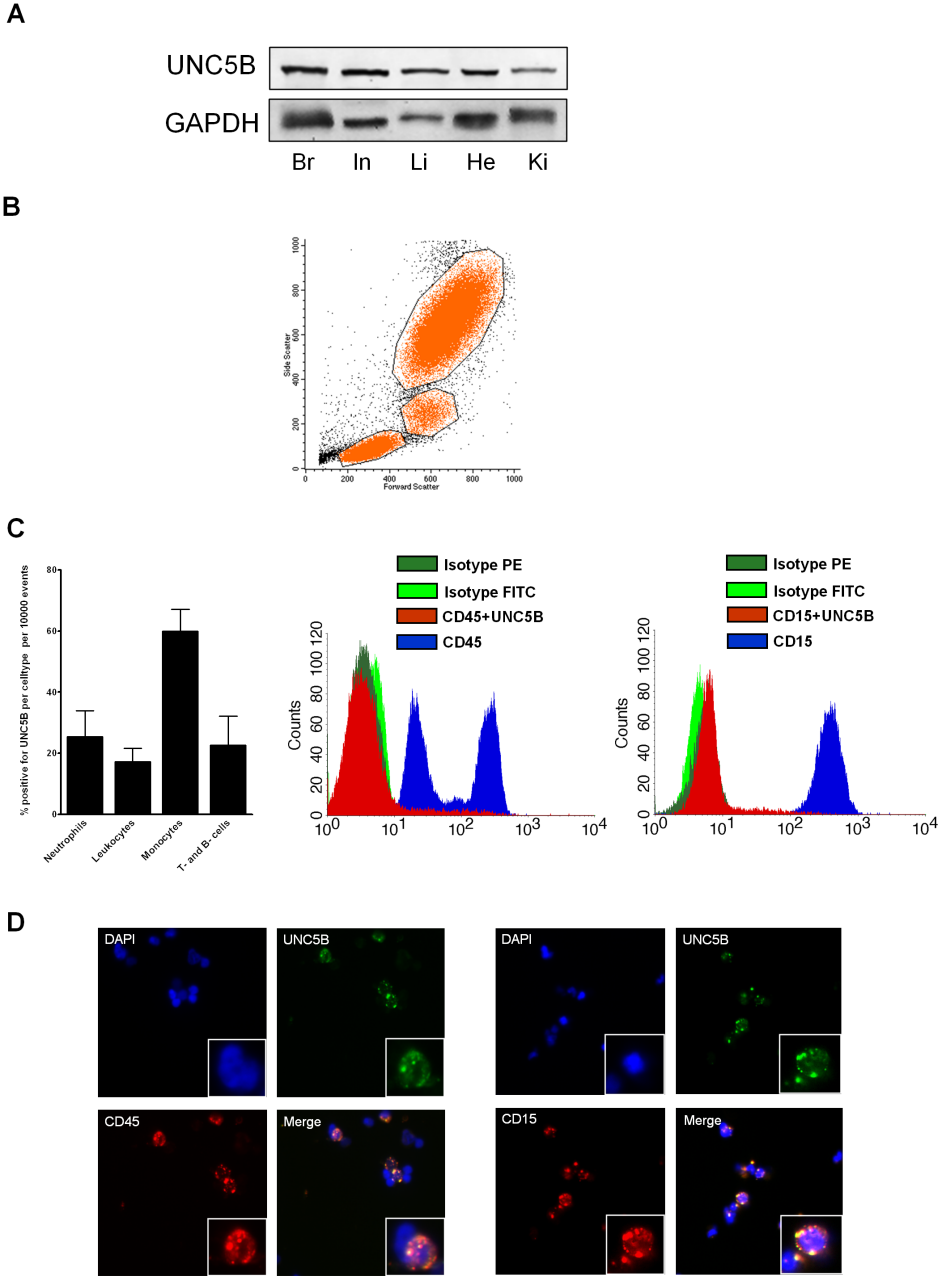
UNC5B expression in murine tissue. **A)** Relative protein expression in murine brain (Br), intestine (In), liver (Li), heart (He) and kidney (Ki) by Western Blot analysis (n = 3; pooled samples). **B)** Gating of leukocytes subpopulations such as lymphocytes, monocytes and neutrophil granulocytes. **C)** Percentage of positive gated neutrophil granulocytes, leukocytes, monocytes and T- and B-cells (n = 5). Representative histograms of UNC5B expression (red) of CD45+ leukocytes and CD15+ neutrophils are shown **D)** Immunofluorescence UNC5B (green = Alexa 488) co-staining on neutrophil leukocytes (CD45 marked) and granulocytes (CD15 marked). Merged images show the localization of UNC5B on the neutrophils and leukocytes (n = 3 per group).

We found significant expression of UNC5B in several murine tissues (Figure 1A). FACS analysis of whole blood showed the expression of UNC5B on the surface of CD15 positive neutrophil, CD45 positive leukocyte (Figure 1C), CD14 positive monocyte and CD3 and CD19 positive T- and B-cell (Figure S1 A and B) population, which we confirmed by immunofluorescent staining (Figure 1D and Figure S1 A and B). To exclude unspecific staining we used IgG and IgM isotype antibodies and corresponding negative controls (Figure S2 A).

### Blocking of the UNC5B Receptor Diminished Transendothelial Migration in vitro

**Figure 2:**
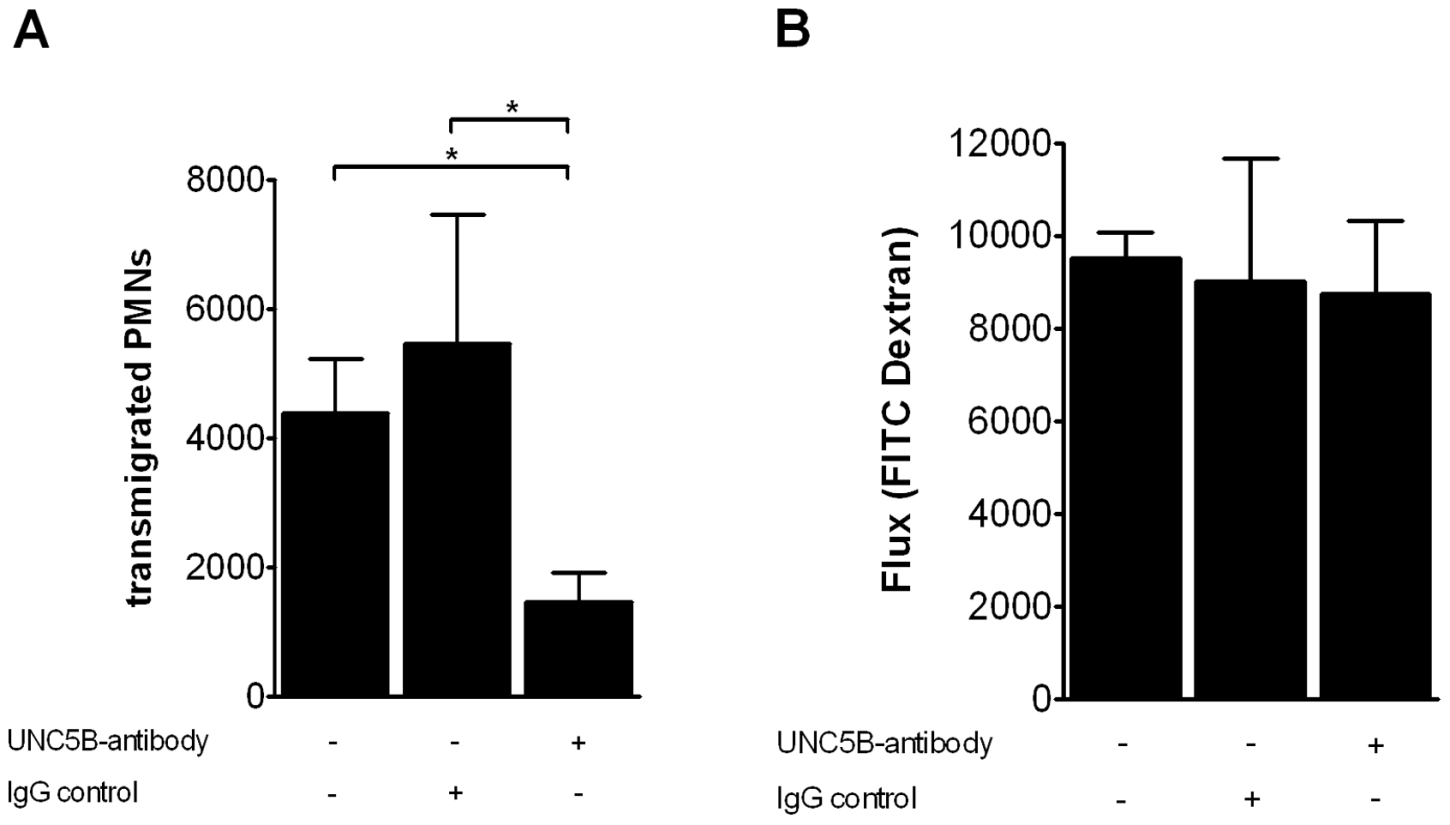
Transendothelial neutrophil migration is inhibited by anti- UNC5B antibody. **A**) PMN transmigration studies were performed in transwells using fMLP as chemoattractant in the lower compartment. PMNs were pre-incubated with either anti-UNC5B antibody or IgG control prior to transmigration. Number of transmigrated PMNs was assessed by MPO measurement. **B**) Passive flux of FITC Dextran across HMEC endothelium monolayers. Flux was detected by measurement of passed FITC Dextran in the basal compartment (n = 6 per condition; *P<0.05 as indicated).

During inflammation leukocytes are recruited to injured tissues where they contribute to local tissue damage [14], [15]. To address the capacity of UNC5B to interfere with leukocyte migration, we blocked the UNC5B receptor on the surface of PMNs. Following this we found a significant reduction in transendothelial migration of PMNs when treated with anti-UNC5B antibody compared to isotype IgG treated PMNs (Figure 2A). When assessing paracellular permeability, we did not detect a significant difference in FITC-Dextran flux when exposing endothelial monolayers to an anti-UNC5B antibody (Figure 2B).

### UNC5B^+/−^ Mice Display Decreased Myocardial IR Injury

**Figure 3:**
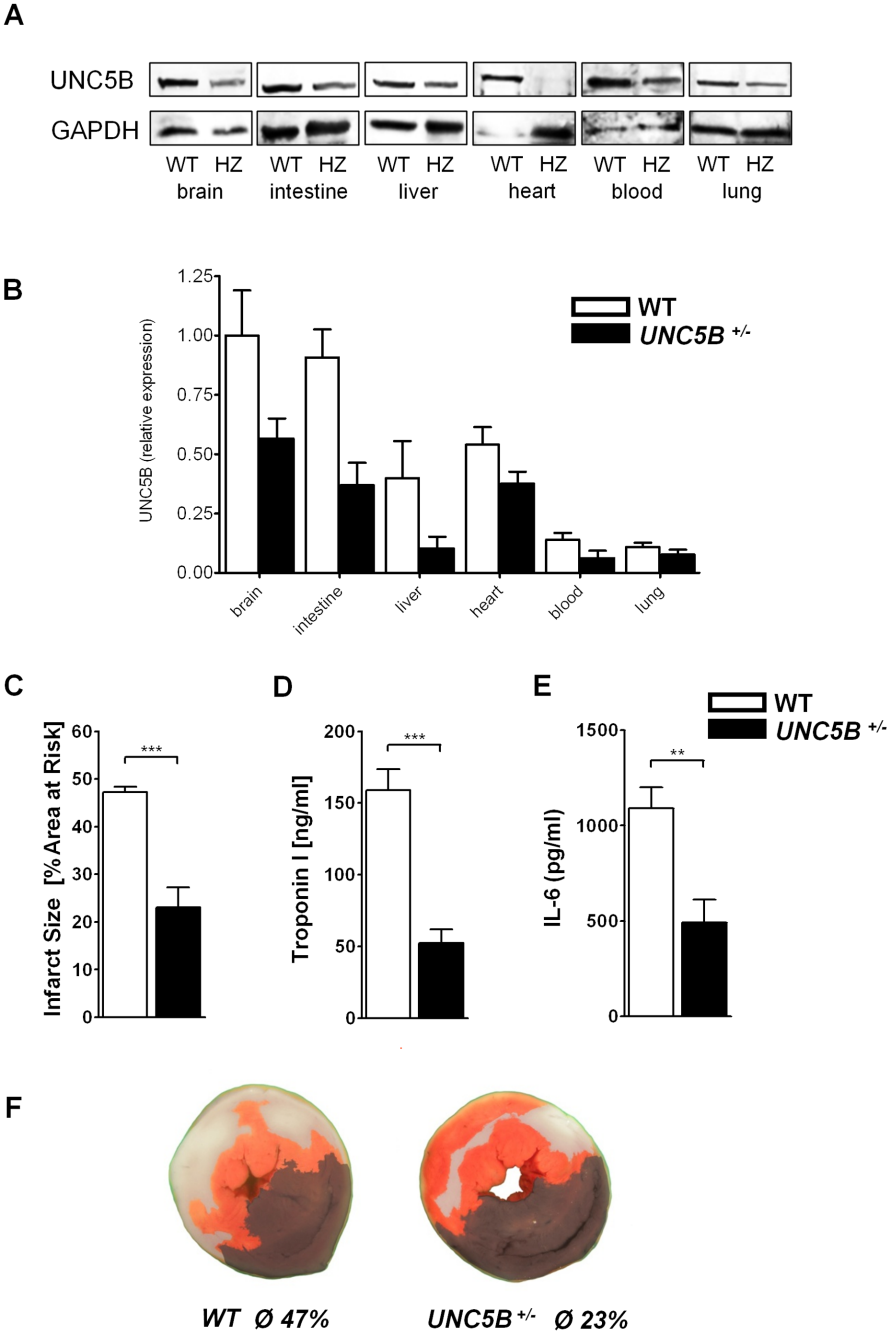
Myocardial infarction is diminished in *UNC5B^+/−^* mice. **A)** Comparison of UNC5B protein expression in *UNC5B^+/−^* and WT animals in different organs (n = 5 per group). **B)** Comparison of UNC5B transcriptional mRNA levels of organs normalized to brain tissue of WT animals (n = 5 per group). **C)** Infarct size in *UNC5B^+/−^* mice compared with WT mice after 60 minutes of myocardial ischemia followed by 2 hours reperfusion. Calculated is the percentage of necrotic tissue to AAR. **D)** and **E)** Correlating serum troponin I and IL-6 levels of *UNC5B^+/−^* and WT mice (n = 6 per group; *P<0.05; **P<0.01 as indicated) **F)** Representative TTC stained heart slices of myocardial infarcts (blue/dark = retrograde Evans blue staining; red and white = AAR; white = infarcted tissue).

As homozygous UNC5B knockout mice are not viable we employed heterozygous *UNC5B^+/−^* mice for our next experiments. First we screened and validated the reduced UNC5B expression levels in several organs in *UNC5B^+/−^* mice (Figure 3A and B). Following myocardial ischemia reperfusion injury infarct sizes were significantly diminished in *UNC5B^+/−^* (23±4% of AAR) mice compared to WT animals (47±1%). Troponin I and IL-6 serum levels corroborated this finding (Figure 3C, D, E and F). This was also associated with decreased infiltration of neutrophils into ischemic areas of *UNC5B^+/−^* mice compared to WT controls (Figure S3).

### UNC5B repression by siRNA Attenuates Myocardial Ischemia Reperfusion Injury

**Figure 4:**
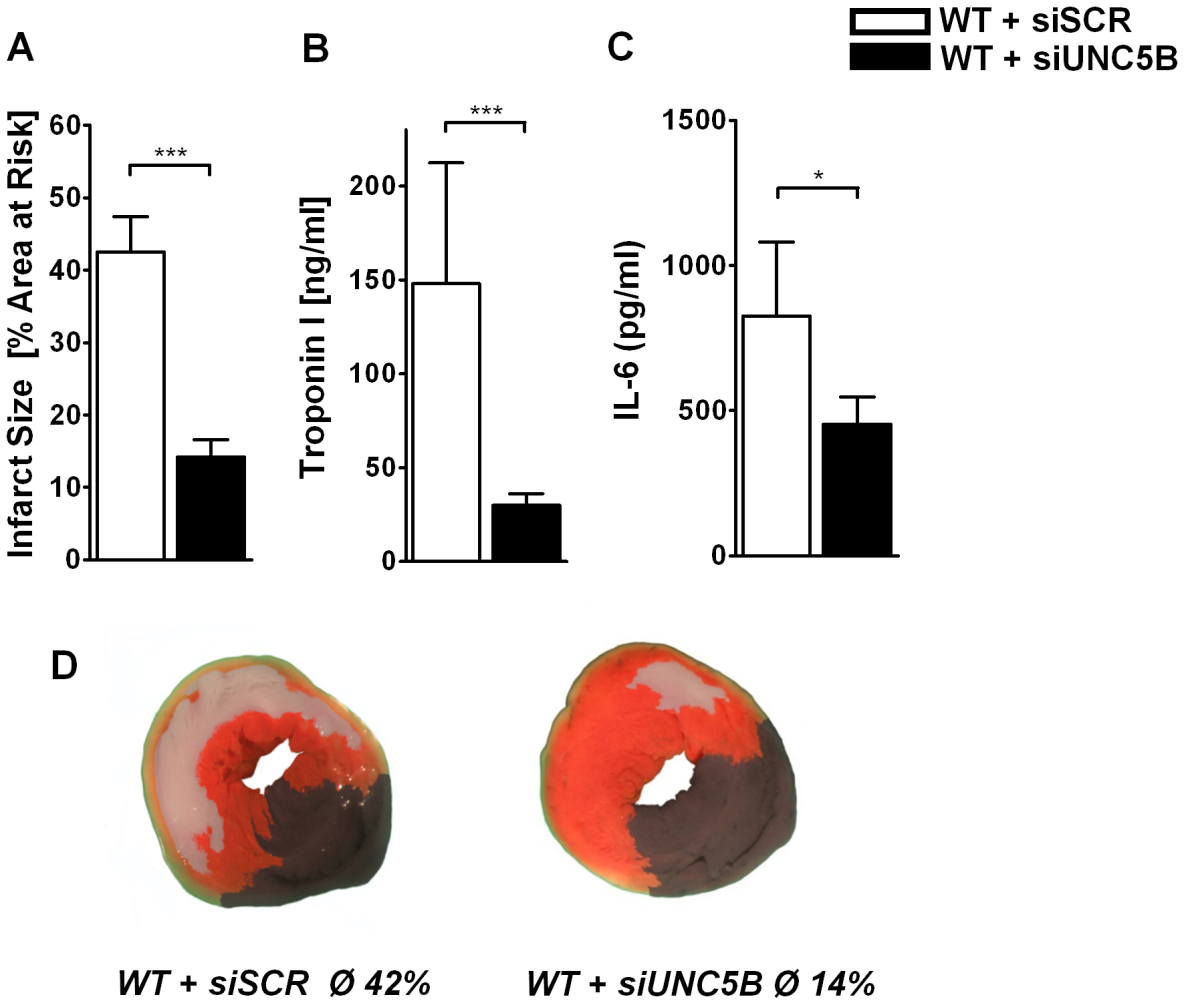
SiRNA in-vivo knockdown of UNC5B attenuates myocardial IR injury in WT mice. **A)** Infarct size of mice with UNC5B specific siRNA treatment compared to mice received non-targeting siRNA after 60 minutes of myocardial ischemia followed by 2 hours reperfusion. Calculated is the percentage of necrotic tissue to AAR. **B)** and **C)** Correlating serum Troponin I and IL-6 levels of these mice (n = 6 per group; *P<0.05; ***P<0.001 as indicated). **D)** Representative TTC stained heart slices of myocardial infarcts (blue/dark = retrograde Evans blue staining; red and white = AAR; white = infracted tissue) of the siRNA treated mice.

For further genetic validation of these previous results and to strengthen our findings we decided to repress UNC5B though siRNA in vivo. 24 h prior to the model of myocardial ischemia we treated WT mice with siRNA intravenously against UNC5B (siUNC5B; 2.5 µg/g body weight) or non targeting siRNA (siSCR; 2.5 µg/g body weight). As for transfection we performed western blot analysis for UNC5B expression. UNC5B was clearly reduced in all siUNC5B compared to siSCR treated mice (Figure S4). Following myocardial IR siUNC5B (14±2% of AAR) treated mice demonstrated a robust infarct size reduction compared to siSCR (42±4% of AAR; Figure 4A) treated animals. This was also reflected in serum troponin I levels (Figure 4B) and reduced levels of IL-6 (Figure 4C and D).

### Functional Inhibition of UNC5B Dampens Myocardial IR Injury

**Figure 5:**
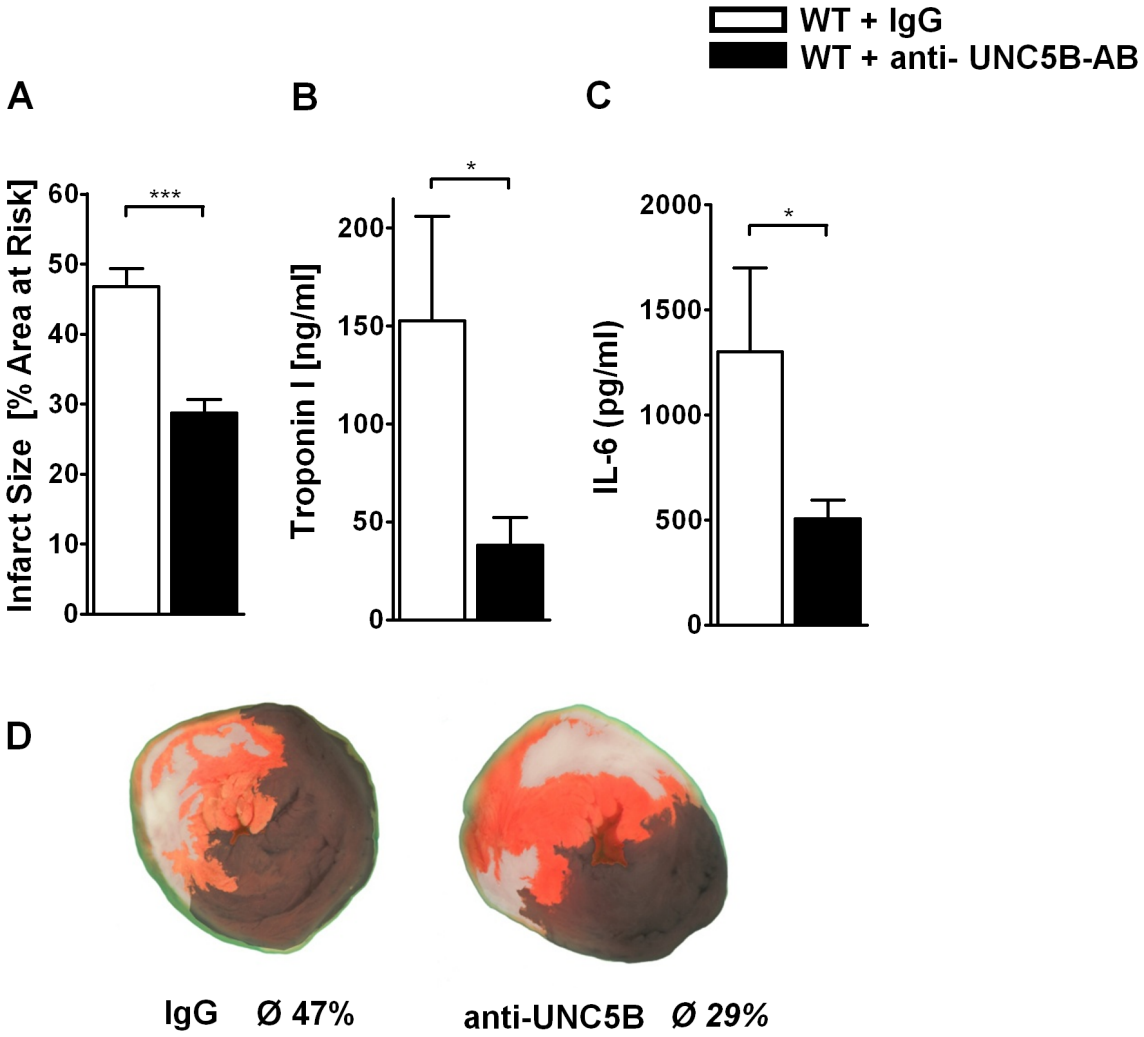
Functional inhibition of UNC5B reduces damage in myocardial IR. **A**) Infarct size after IR in WT mice receiving antibody against UNC5B (2.5 µg/mouse) iv. 30 min before onset of surgery. Controls were treated with corresponding IgG antibody. Calculated is the percentage of necrotic tissue to AAR. **B)** and **C)** Correlating serum Troponin I and IL-6 levels of these mice. (n = 6 per group; *P<0.05; ***P<0.001 as indicated). **D)** Representative TTC stained heart slices of myocardial infarcts (blue/dark = retrograde Evans blue staining; red and white = AAR; white = infracted tissue) of the antibody treated mice.

To gain further insight into the potential role of UNC5B as a therapeutic target we administered 2.5 µg antibody at time point of ischemia and following reperfusion in 100 µl PBS against UNC5B per mouse (WT+UNC5B-AB) 30 minutes prior to ischemia. As control a corresponding IgG antibody in 100 µl PBS was applied in WT mice (WT+IgG control). We found significantly reduced tissue damage in the WT+UNC5B-AB group (29±2% of AAR) compared to WT+IgG control (47±2% of AAR; Figure 5A and D) group. This was corroborated through troponin I (152±53 versus 38±14 ng/ml; Figure 5B) and IL-6 (1302±399 versus 507±87 ng/ml; Figure 5C) levels.

**Figure 6:**
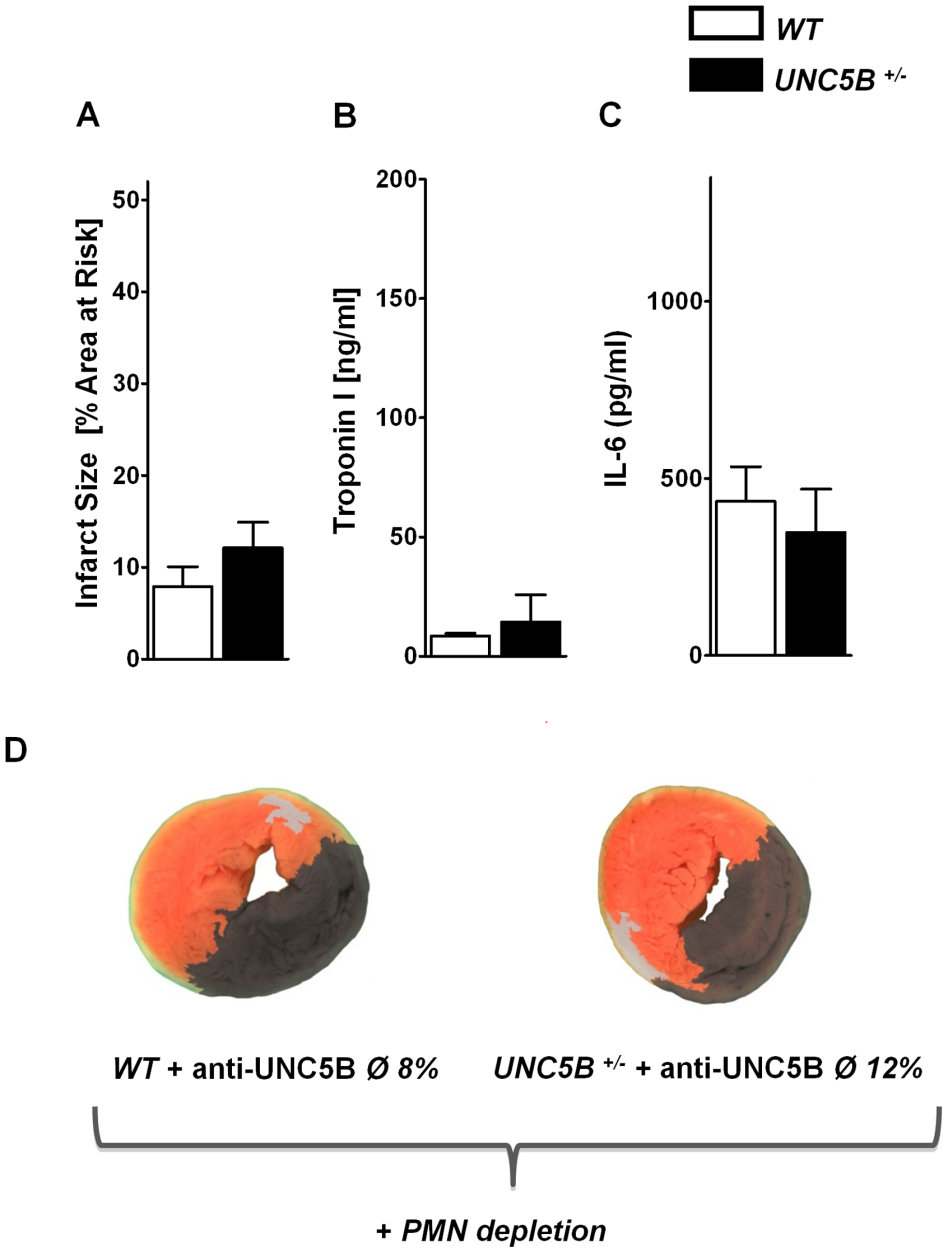
Functional inhibition of UNC5B after depletion of neutrophil granulocytes does not results not in additional cardioprotection. **A)** Infarct size following IR in WT and UNC5B^+/−^ mice after neutrophil granulocyte depletion and subsequent anti- UNC5B antibody adminsitration. Calculated is the percentage of necrotic tissue to AAR. **B)** and **C)** Correlating serum Troponin I and IL-6 levels of these mice. (n = 4 per group). **D)** Representative TTC stained heart slices of myocardial infarcts (blue/dark = retrograde Evans blue staining; red and white = AAR; white = infracted necrotic tissue.

We then moved on to determine whether the observed effect of UNC5B inhibition could be influenced through depletion of neutrophils prior to myocardial IR. Following depletion of neutrophils through Ly-6G antibody injection we again performed the above described experiment. We did not observe a reduction of infarct size in neither WT nor *UNC5B^+/−^* mice through the injection of an anti- UNC5B antibody (Figure 6).

## Discussion

UNC5B, which is also named UNC5H2, was initially described as a receptor for the neuronal guidance protein netrin-1 in the CNS [16]–[18]. Recent data have however shown that UNC5B also holds significant function in the immune system in particular during immune cell migration and the orchestration of an acute inflammatory response [8], [9], [19]–[21]. As such we hypothesized that UNC5B might also hold relevance during myocardial IR and report here that UNC5B significantly affects the extent of myocardial IR injury. In addition, we found that functional inhibition of UNC5B and the repression of UNC5B through siRNA significantly attenuates the extent of myocardial IR injury.

In initial experiments we found robust expression of UNC5B in a variety of murine tissues. The inhibition of UNC5B reduced transendothelial migration of leukocytes in vitro. This in vitro finding translated into reduced reperfusion injury in UNC5B^+/−^ animals with a significant reduction blood serum troponin I levels. The diminished tissue destruction was associated with decreased PMN infiltration within the infarcted tissue. Since cell migration, adhesion and survival are critical characteristics influencing acute inflammatory processes it might be expected that UNC5B could influence the control of leukocyte infiltration, tissue inflammation and as such reperfusion injury. Yet, controversial data exist about the role of UNC5B on leukocyte migration. Recent studies employing the UNC5B ligand netrin-1 showed that netrin-1 reduces the infiltration of immunocompetent cells such as PMNs and monocytes in an UNC5B dependent manner [8], [21]. Treatment with an anti-UNC5B antibody abolished the effects of netrin-1 during fMLP-induced chemotaxis in vitro and in a model of renal ischemia reperfusion in vivo [22]. In a study by Mirakaj et al. the investigators showed that the anti-inflammatory effect of netrin-1 during Zymosan A induced peritonitis might be mediated by the adenosine 2B (A2BAR) receptor [23]. This is supported by data of Aherne et al. who showed that UNC5B blockade was not able to affect DSS induced colitis but was A2BAR receptor dependent [24]. These findings however do not stand in contrast to our presented data. Our data presented here indicate that UNC5B acts as a pro-inflammatory guidance receptor that facilitates the infiltration of leukocytes into sites of inflammation and as such influences the extent of tissue injury following IR. Whether netrin-1 uncovers its anti-inflammatory potential through UNC5B, the A2BAR or another receptor was not subject to this study and needs to be investigated further elsewhere. It is however well established that UNC5B seems to serve as pro-migratory receptor in a broader context [4]. Our data confirm this hypothesis during reperfusion injury and show that UNC5B holds significant impact on tissue injury associated with infiltration of leukocytes into the affected tissue site. This is especially corroborated by the finding, that following depletion of neutrophils the injection of an anti-UNC5B antibody did not result in a further protective effect for the ischemic myocardial tissue.

We found that functional inhibition of UNC5B in mice before the onset of myocardial ischemia was protective against myocardial tissue injury. These findings contribute to affirm our hypothesis that UNC5B might be a therapeutic target for myocardial IR injury in the future. Intervention using UNC5B antibody was highlighted previously in a study by Tadagavadi et al. during renal ischemia followed by reperfusion [21]. In this study the investigators demonstrated that UNC5B inhibition resulted in a reversal of the protective effect of netrin-1. A crucial difference between our and previous studies employing anti-UNC5B antibodies is that the time-point, the way of the antibody administration and the dose of antibody used. We performed i.v. injection of the anti-UNC5B antibody 30 minutes prior to ischemia. The dose we employed was 2.5 µg/mouse (125 µg/kg body weight). In contrast to that Tadagavadi et al. injected the anti-UNC5B antibody i.p. 18 hours before the induction of ischemia in a dose of 800 µg/kg body weight. Aherne et al. even started the i.p. administration of the UNC5B blocking antibody 2 days prior to the induction of DSS colitis [24]. The dose of the UNC5B blocking antibody used in this study was also 800 µg/kg body weight. In an effort to come close to clinical practice we decided to use the time point of antibody injection close to the institution of myocardial ischemia. This might explain the differences in the findings from our study to these previously published data since as the time-point, the route of administration and the dose of the UNC5B blocking antibody differed significantly between all these studies.

Taken together the results of our study demonstrate that UNC5B significantly contributes to the pathology observed during myocardial IR injury. The repression and functional inhibition of UNC5B prevented cardiac tissue destruction during reperfusion injury. As such UNC5B repression might be a potential target for a possible therapeutic intervention during myocardial IR injury in the future.

## Supporting Information

Figure S1 **UNC5B expression on human monocytes and lymphocytes. A)** UNC5B expression on leukocytes assessed through flowcytometry and immunhistochemical staining using CD14 as monocyte marker and **B)** UNC5B expression on lymphocytes assessed through flowcytometry and immunhistochemical staining using CD3+ CD19 as specific lymphocytes marker. (n = 3 per group).
(TIF)

Figure S2 **Isotype and secondary antibody immunofluorescence control staining. A)** Representative isotype IgG, IgM control and negative control staining of UNC5B and the blood cell types monocytes, neutrophil granulocytes and leukocytes in summary are displayed.
(TIF)

Figure S3 **Neutrophil infiltration in infarcted heart tissue of WT and** ***UNC5B^+/−^*** **mice. A)** Representative sections of AAR heart tissue in WT and UNC5B*^+/−^* heterozygous animals after 60 min ischemia followed by 120 min reperfusion. Heart tissue was stained using anti-troponin antibody (green; Alexa 488). Neutrophils were stained using anti-neutrophil antibody (red; Alexa 594; n = 3 per group). **B)** Neutrophil count in tissue sections of the correlating groups described in A). (n = 30 sections per group). **C)** Images of IgG control staining and negative control staining are displayed.
(TIF)

Figure S4 **UNC5B expression in WT animals following siRNA injection. A)** Westernblot analysis of heart tissue of WT animals 24 h post siUNC5B or siSCR ( = nontargeting siRNA) injection **B)** Correlating Westernblot analysis of blood samples of these mice (n = 4 per group).
(TIF)
