# Supplementary figures and images for: The Uncoordinated-5 Homolog B (UNC5B) Receptor Increases Myocardial Ischemia-Reperfusion Injury
Source: PLoS One. 2013 Jul 23;8(7):e69477. doi: 10.1371/journal.pone.0069477 (PMC3720625; doi:10.1371/journal.pone.0069477)

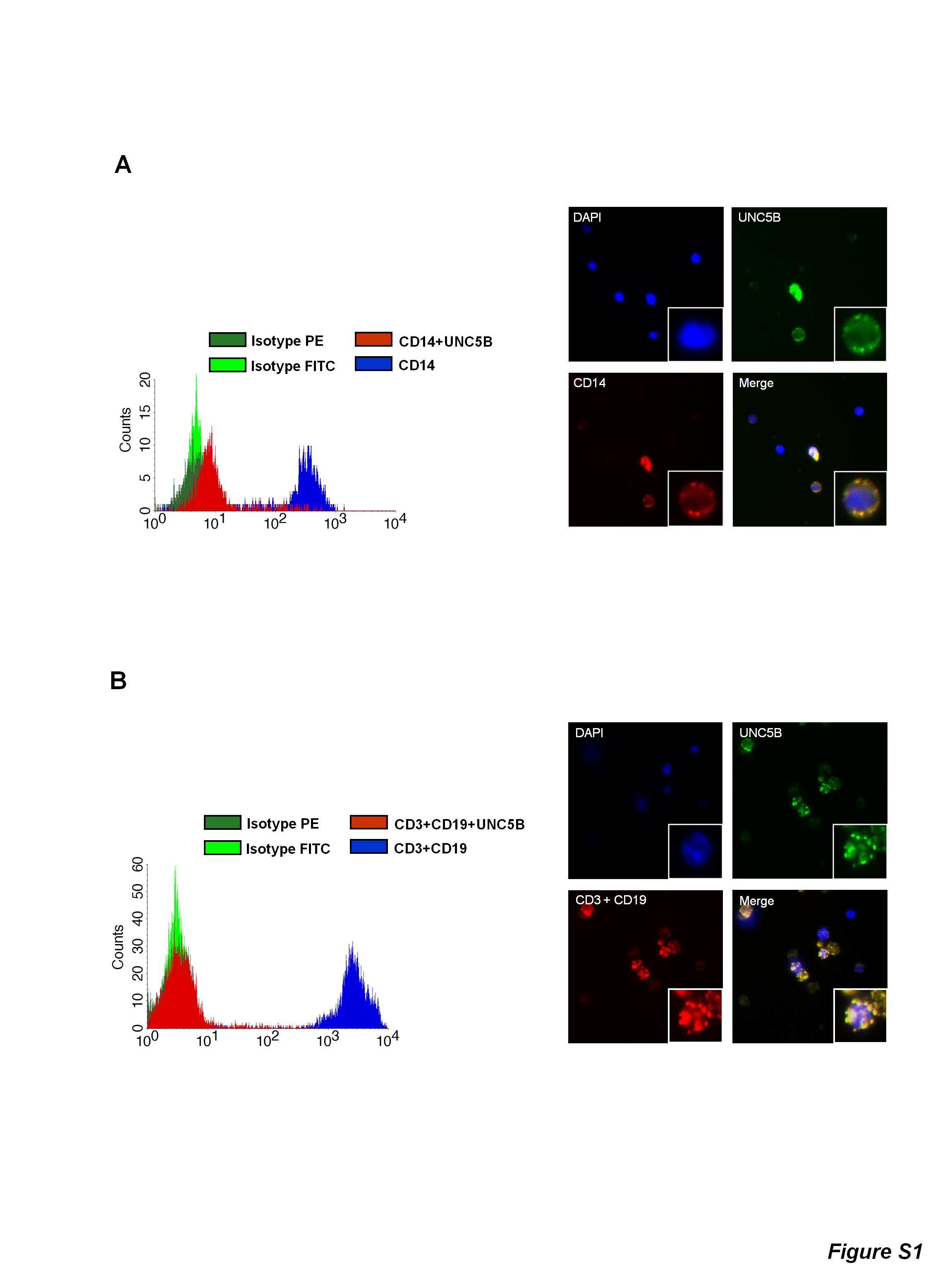

Supplement: Figure S1 — UNC5B expression on human monocytes and lymphocytes. A) UNC5B expression on leukocytes assessed through flowcytometry and immunhistochemical staining using CD14 as monocyte marker and B) UNC5B expression on lymphocytes assessed through flowcytometry and immunhistochemical staining using CD3+ CD19 as specific lymphocytes marker. (n = 3 per group). (TIF) [file pone.0069477.s001.tif]

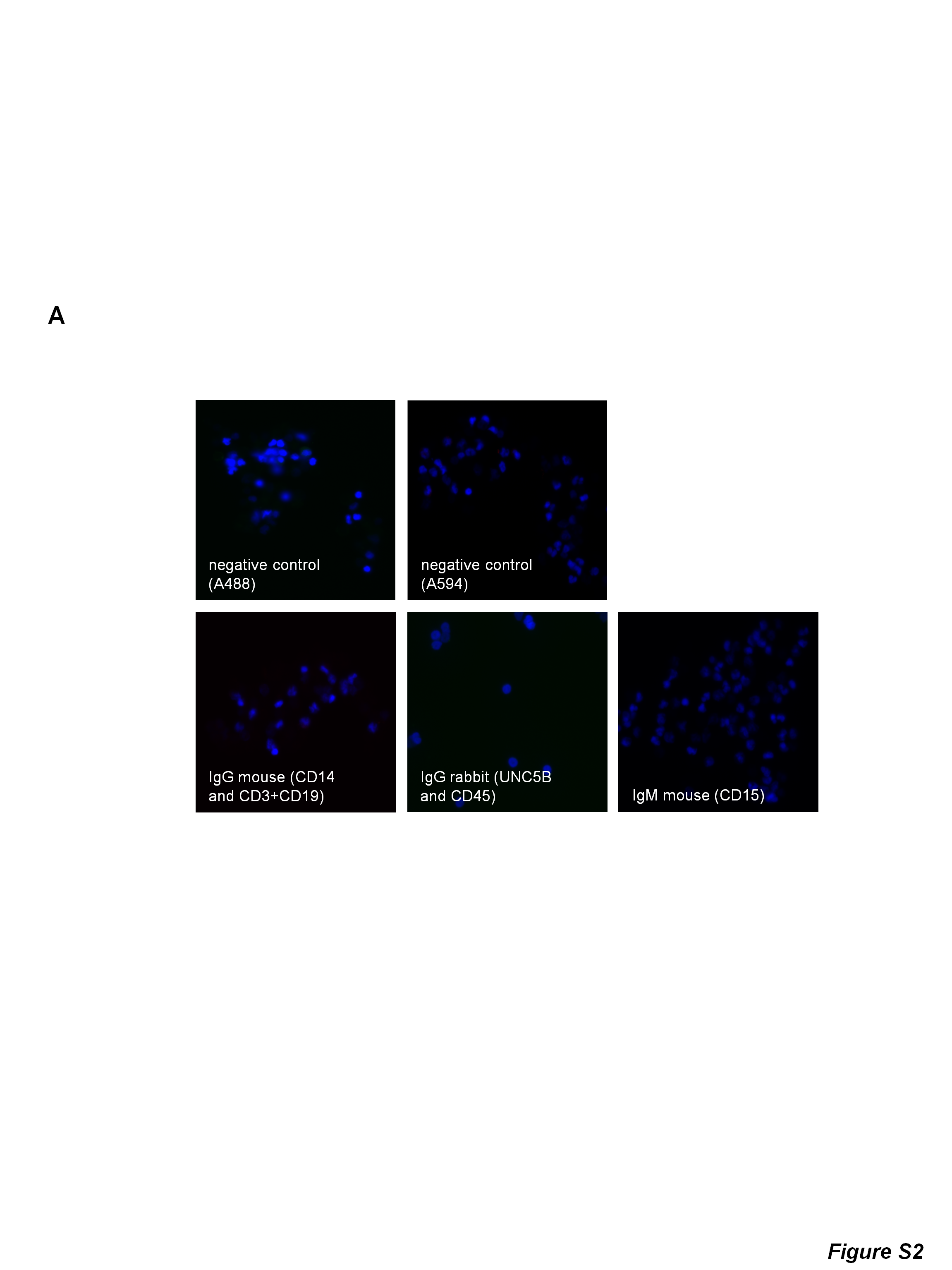

Supplement: Figure S2 — Isotype and secondary antibody immunofluorescence control staining. A) Representative isotype IgG, IgM control and negative control staining of UNC5B and the blood cell types monocytes, neutrophil granulocytes and leukocytes in summary are displayed. (TIF) [file pone.0069477.s002.tif]

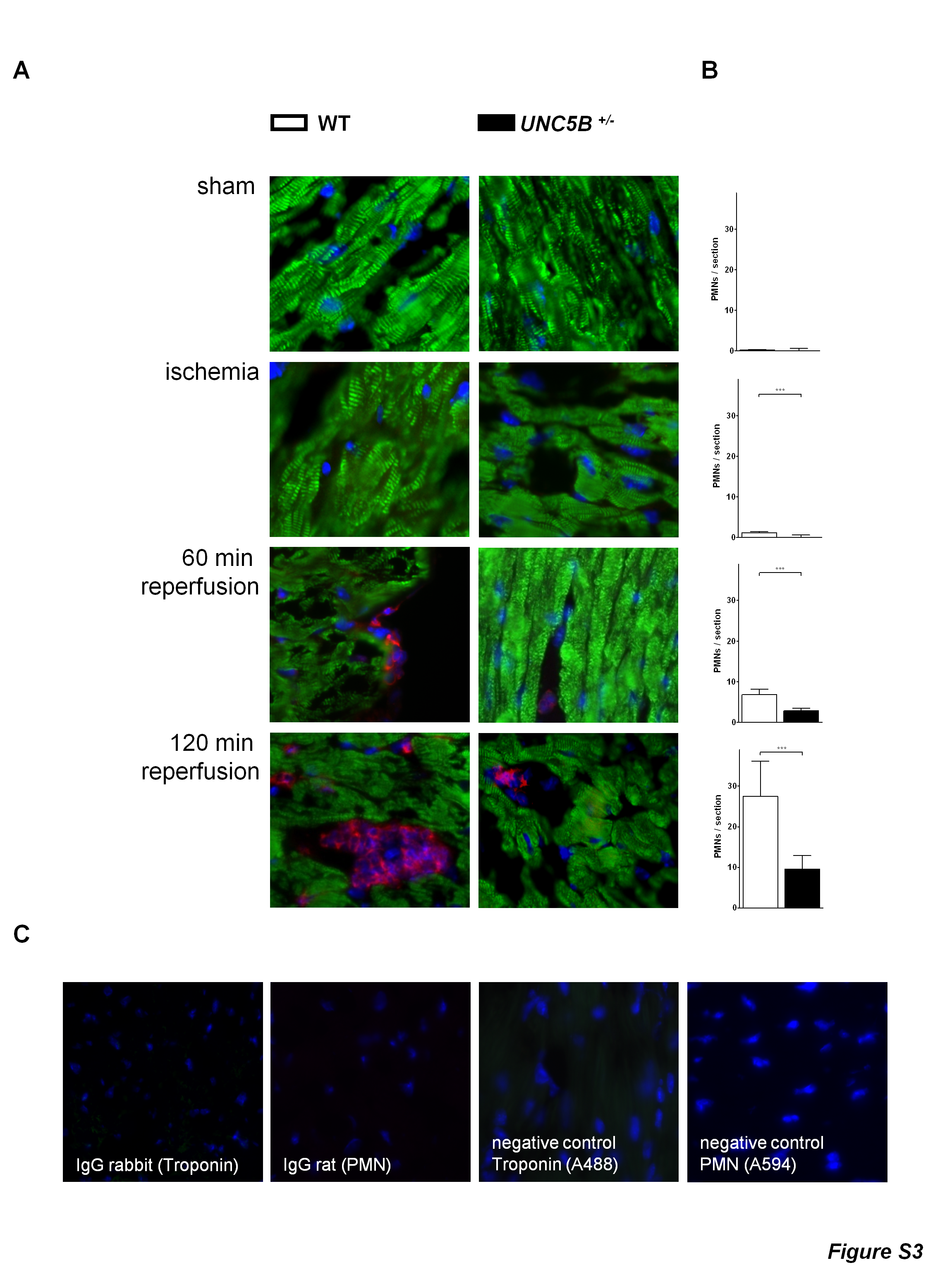

Supplement: Figure S3 — Neutrophil infiltration in infarcted heart tissue of WT and UNC5B+/− mice. A) Representative sections of AAR heart tissue in WT and UNC5B+/− heterozygous animals after 60 min ischemia followed by 120 min reperfusion. Heart tissue was stained using anti-troponin antibody (green; Alexa 488). Neutrophils were stained using anti-neutrophil antibody (red; Alexa 594; n = 3 per group). B) Neutrophil count in tissue sections of the correlating groups described in A). (n = 30 sections per group). C) Images of IgG control staining and negative control staining are displayed. (TIF) [file pone.0069477.s003.tif]

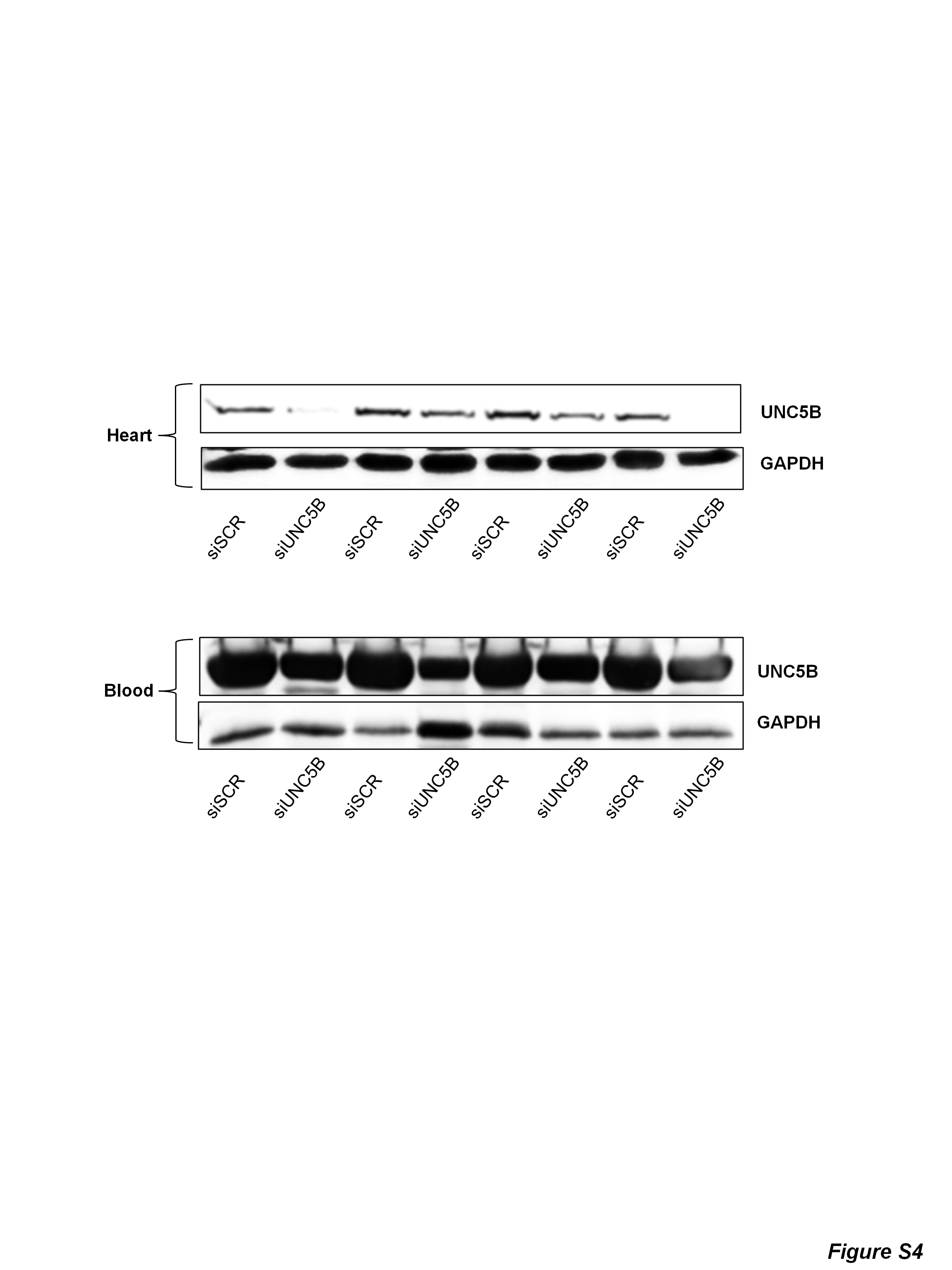

Supplement: Figure S4 — UNC5B expression in WT animals following siRNA injection. A) Westernblot analysis of heart tissue of WT animals 24 h post siUNC5B or siSCR ( = nontargeting siRNA) injection B) Correlating Westernblot analysis of blood samples of these mice (n = 4 per group). (TIF) [file pone.0069477.s004.tif]
